# Supplementary material for: NCX1 reverse mode promotes calcium-dependent Neutrophil Extracellular Trap formation and lung damage in chronic obstructive pulmonary disease
Source: Nat Commun. 2026 Mar 11;17:3801. doi: 10.1038/s41467-026-69636-1 (PMC13111594; doi:10.1038/s41467-026-69636-1)
Supplement: Supplementary file 1 — Supplementary Information [file 41467_2026_69636_MOESM1_ESM.pdf]

Supplementary Information for

NCX1 reverse mode promotes calcium-dependent NETs formation and lung damage in chronic obstructive pulmonary disease

Liao et al.

Supplementary Information included:

Supplementary Fig.1-Supplementary Fig.4
Supplementary Table 1-Supplementary Table 4

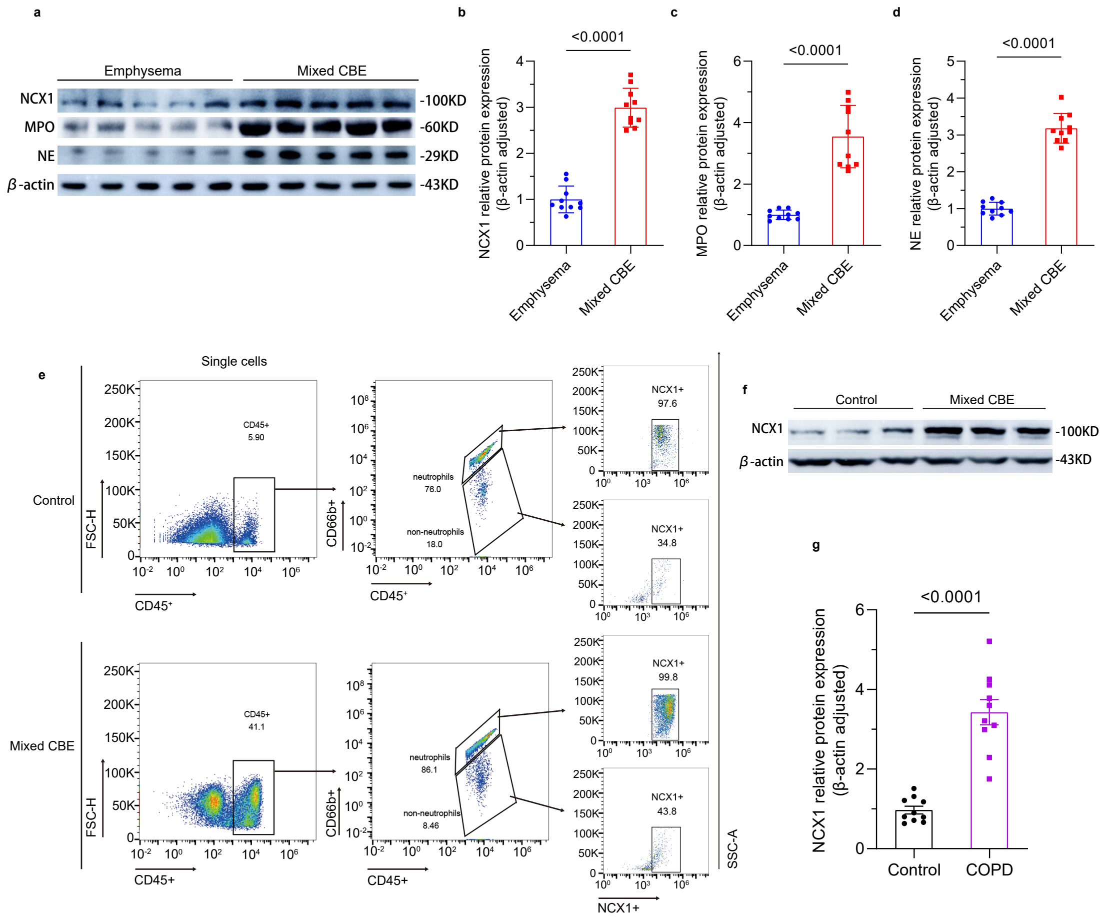

Supplementary Fig. 1 NXC1 expression in lung tissues and peripheral blood of Mixed CBE patients.

a-d WB analyzes protein levels of NCX1 (**b**), MPO (**c**) and NE (**d**) in lung tissues from Emphysema patients (n = 10 individuals) and Mixed CBE patients (n = 10 individuals), normalized to β-actin and displayed relative to controls. **e** Flow cytometry identifies NCX1 expression in BALF-derived neutrophils relative to other immune cell populations in Control and Mixed CBE patients (n = 5 individuals). **f-g** WB detects NCX1 protein levels in peripheral blood neutrophils from Control and Mixed CBE patients (n = 5 individuals). Each data point represents one biologically independent replicate with two technical replicates (**b-d)**, one biologically independent replicate without technical replicates (**g**). All quantitative data are presented as Mean ± SD. Two-sided t-test was used to calculate the p values (**b-d, g**). At least 3 times each experiment was independently repeated with similar results. Source data are provided as a Source Data file. CBE, chronic bronchitis emphysema; MPO, myeloperoxidase; NE,

neutrophil elastase; Mixed, chronic obstructive pulmonary disease.

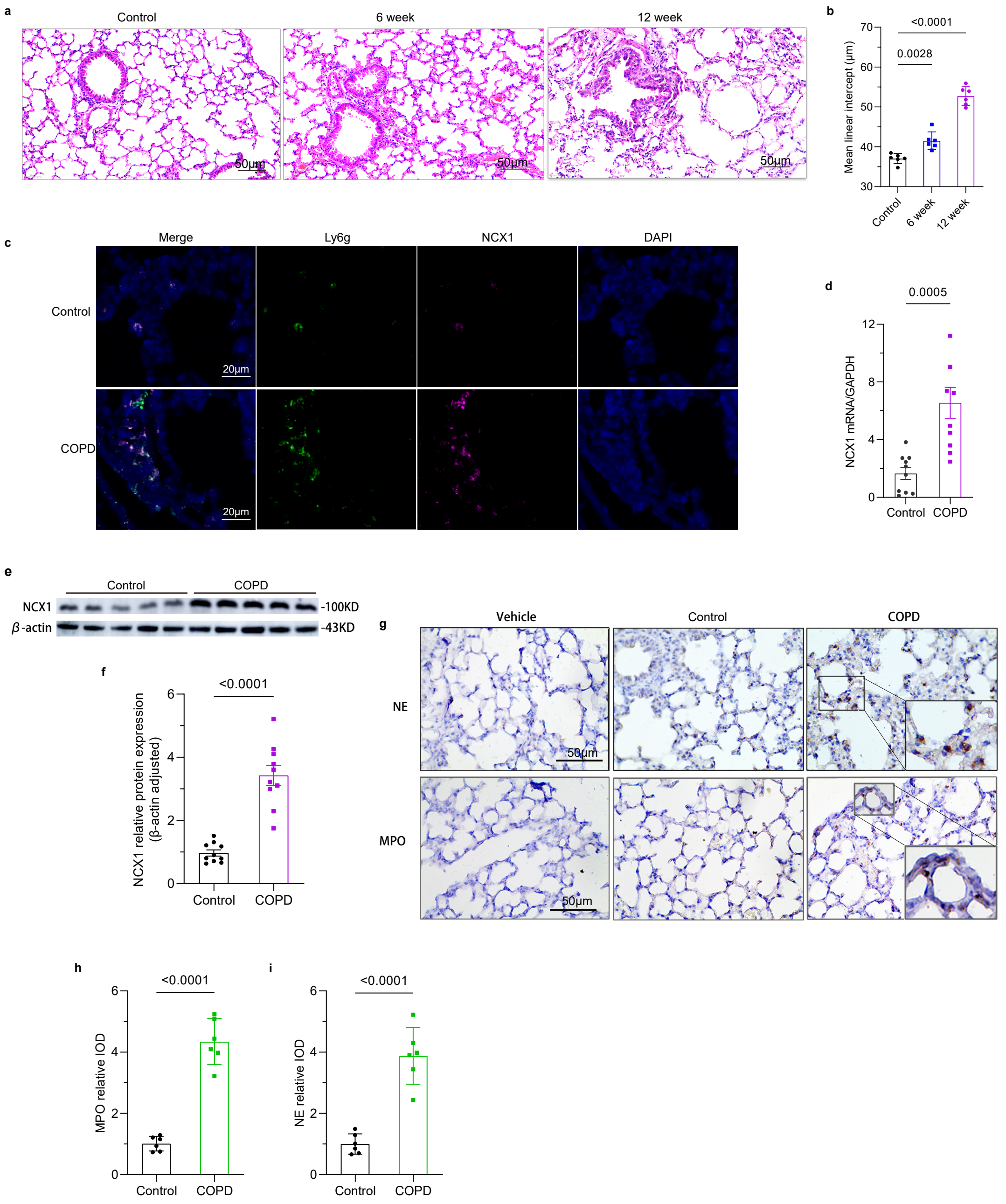

Supplementary Fig. 2 NCX1 elevation and activation in COPD mice.

a Representative H&E-stained lung sections showing alveolar destruction and airway inflammation in mice subjected to CS exposure for 6 weeks and 12 weeks. Scale bar = 50 μm. **b** Quantification of mean linear intercept values in Control and CS-exposed mice (*n* = 6 mice). **c** Representative immunofluorescence images show NCX1 (purple) expression and its co-localization with neutrophils (Ly6g^{+}, green) in lung tissues from Control and COPD mice (*n* = 5 mice). Blue, DAPI-stained nuclei. Scale bar = 20 μm.

d RT-qPCR analyzes mRNA levels of NCX1 in lung tissues from Control and COPD mice (*n* = 10 mice). **e-f** WB analysis of NCX1 protein levels in lung tissues from Control and COPD mice (*n* = 10 mice), normalized to β-actin and displayed relative to controls. **g-i** Immunohistochemical staining shows the protein expression of NE and MPO in lung tissues of Control and COPD mice (*n* = 6 mice). Scale bar = 50 μm. Each data point represents one biologically independent replicate with three technical replicates (**b, d)**, one biologically independent replicate with two technical replicates (**f**), one biologically independent replicate (**h, i**). All quantitative data are presented as Mean ± SD. Two-sided *t*-test (**d, f, h, i**) and one-way ANOVA with Tukey's multiple comparison test (**b**) were used to calculate the *p* values. At least 3 times each experiment was independently repeated with similar results. Source data are provided as a Source Data file. MPO, myeloperoxidase; NE, neutrophil elastase; COPD, chronic obstructive pulmonary disease; IOD, integrated optic density.

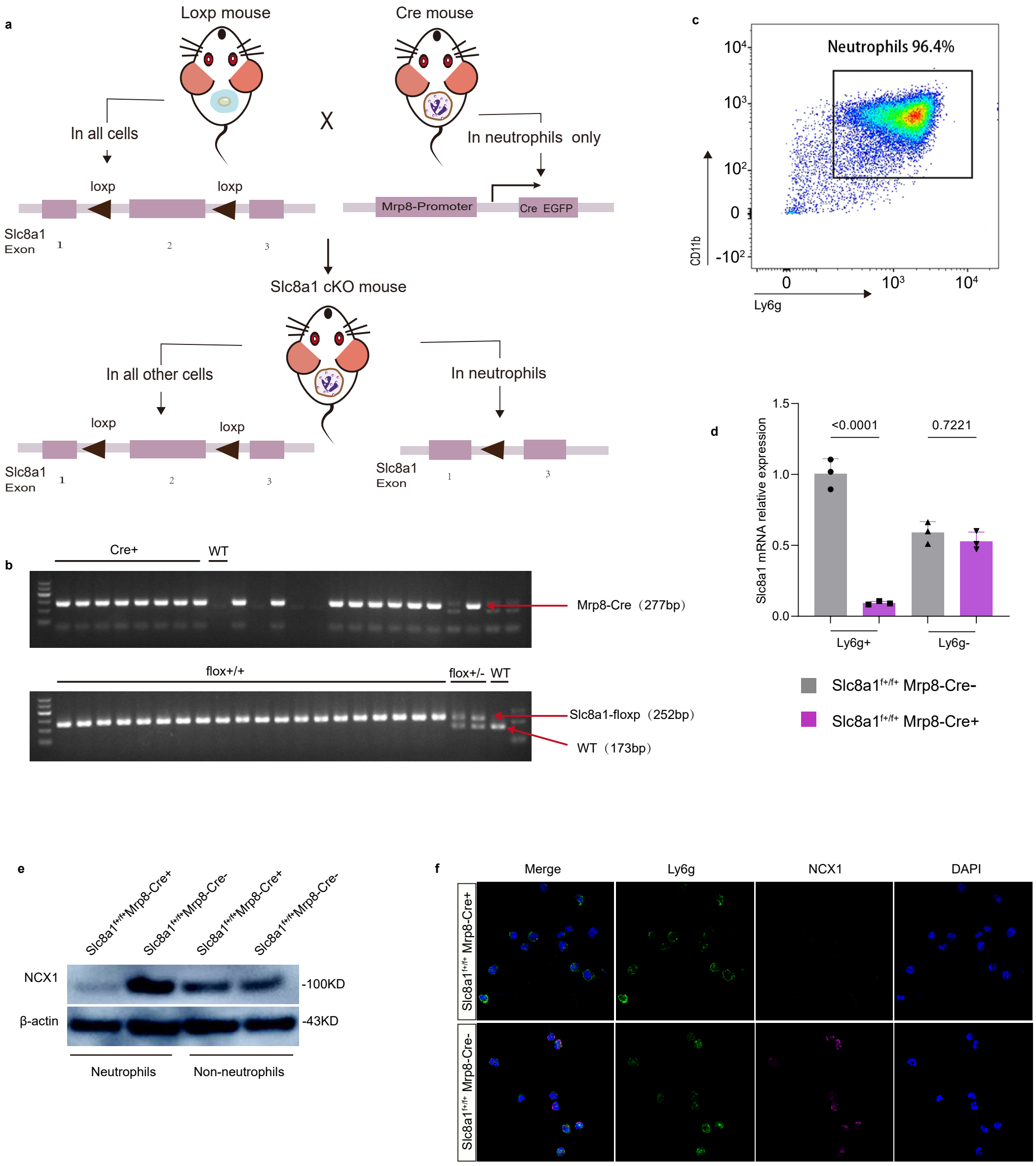

Supplementary Fig. 3 Neutrophilic NCX1 conditional knockout in mice.

a Schematic procedure for the neutrophil-specific *Slc8a1* cKO mice (*Slc8a1*$^{f+/f+}$;Mrp8-Cre^{+}) and Control mice (*Slc8a1*$^{f+/f+}$;Mrp8-Cre^{-}). **b** PCR identifies Mrp-Cre bands and *Sl*c8a1 loxp bands in mice with different genotypes. **c** Bone marrow-derived neutrophils from *Slc8a1*$^{f+/f+}$;Mrp8-Cre^{-} and *Slc8a1*$^{f+/f+}$;Mrp8-Cre^{+} mice were isolated and verified by flow cytometry with 95%-98% purity. **d-e** RT-qPCR and WB detect *Slc8a1* expression in bone marrow-derived neutrophils or non-neutrophils from *Slc8a1*$^{f+/f+}$;Mrp8-Cre^{-} and *Slc8a1*$^{f+/f+}$;Mrp8-Cre^{+} mice (n = 3 mice). **f** Representative immunofluorescent images of NCX1 (purple) and expression in bone marrow-derived neutrophils (Ly6g, green) from *Slc8a1*$^{f+/f+}$;Mrp8-Cre^{-} and *Slc8a1*$^{f+/f+}$;Mrp8-Cre^{+} mice.

Scale bar = 10 μm. Blue, DAPI-stained nuclei. Each data point represents one biologically independent replicate with three technical replicates (**d**). All quantitative data are presented as Mean ± SD. One-way ANOVA with Tukey's multiple comparison test in this figure was used to calculate the *p* values (**d**). At least 3 times each experiment was independently repeated with similar results. Source data are provided as a Source Data file. WT, wild type; cKO, conditional knockout; ns, no significance.

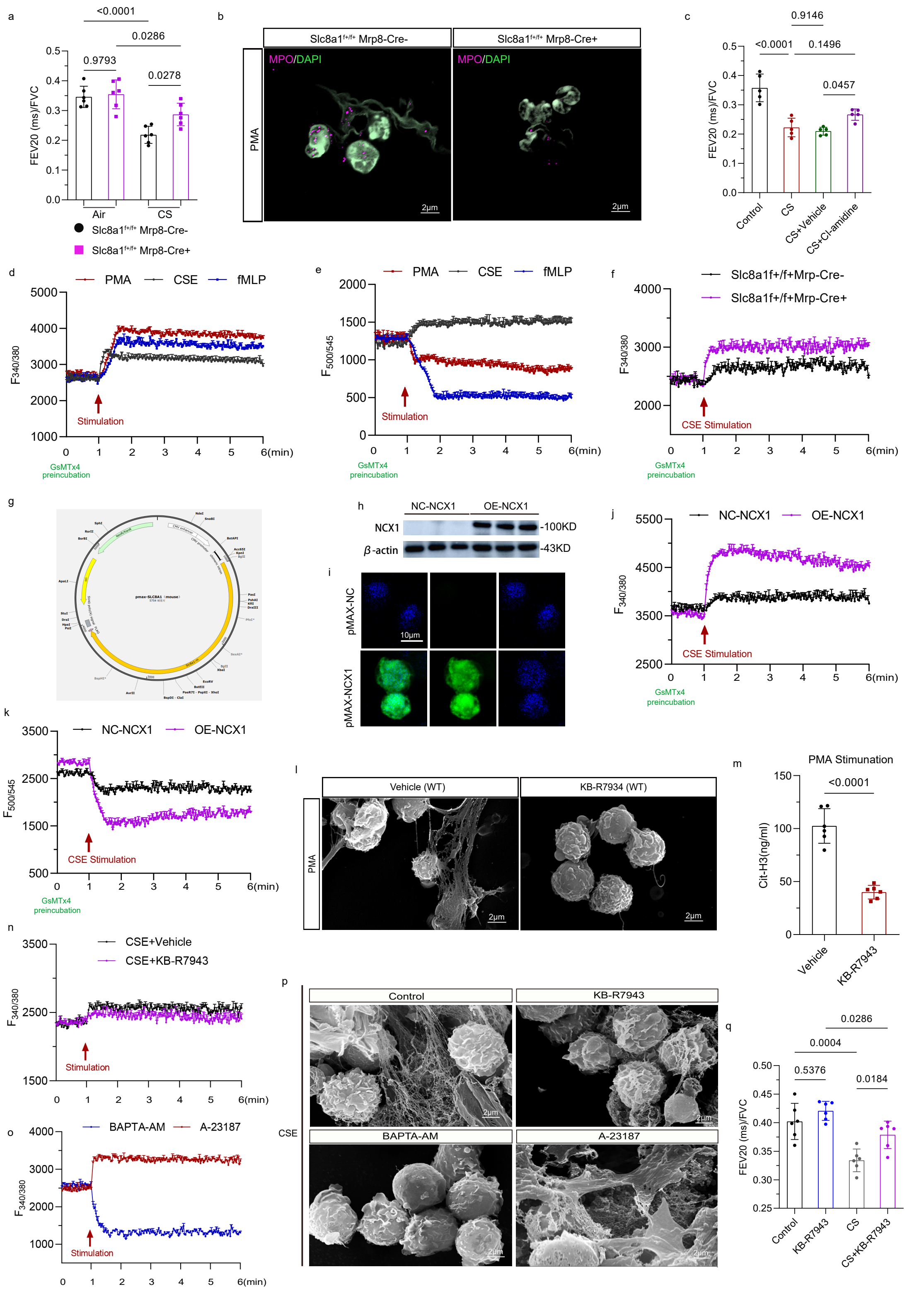

Supplementary Fig. 4. Association of NCX1 reverse mode with Ca^{2+} flux and NETs release

a Pulmonary function parameter $FEV_{20(ms)}/FVC$ were measured after 12-week CS exposure (n = 6 mice). **b** Representative immunostaining images of MPO (purple)

expression in PMA-stimulated bone marrow neutrophils from *Slc8a1*$^{f+/f+}$;Mrp8-Cre^{-} and *Slc8a1*$^{f+/f+}$;Mrp8-Cre^{+} mice. Green, DAPI-stained nuclei. Scale bar = 2 μm. **c** Pulmonary function parameter $FEV_{20(ms)}$/FVC of Control, CS, CS+Vehicle and CS+Cl-amidine mice were measured after 12-week CS exposure (n = 5 mice). **d-e** Simultaneous real-time measurements of intracellular Ca^{2+} (F340/380) and Na^{+} (F500/545) in GsMTx4 pre-incubated neutrophils by fluorescent probes upon stimulation with fMLP, PMA, or CSE (n = 3). **f** Ca^{2+} influx in bone marrow-derived neutrophils (pre-incubated with GsMTx4) from *Slc8a1*$^{f+/f+}$;Mrp8-Cre^{-} and *Slc8a1*$^{f+/f+}$;Mrp8-Cre^{+} mice upon CSE stimulation (n = 3). **g** Plasmid construction of NCX1 overexpression. **h-i** Western blot analysis and immunofluorescence validated the expression of NCX1 in NCX1-transfected CHO-K1 cells and vector controls (n = 3). Blue, DAPI-stained nuclei. Green, NCX1 fluorescence. Scale bar = 10 μm. **j-k** Ca^{2+} and Na^{+} influx in NCX1-transfected CHO-K1 cells and vector controls (pre-incubated with GsMTx4) upon CSE stimulation (n = 3). **l** NETs formation in PMA-stimulated bone marrow-derived neutrophils (WT mice) with Vehicle or KB-R7943 treatment are visualized by scanning electron microscope. Scale bar = 2 μm. **m** ELISA detects Cit-H3 expression in the cell culture of PMA-stimulated bone marrow-derived neutrophils (WT mice) between Vehicle and KB-R7943 treatment groups (n = 6 mice). **n** Ca^{2+} influx detection in CSE-stimulated bone marrow-derived neutrophils (*Slc8a1* cKO mice) between Vehicle and KB-R7943 treatment groups (n = 3 mice). **o** Simultaneous real-time measurements of intracellular Ca^{2+} by fluorescent probes in bone marrow-derived neutrophils (WT mice) with BAPTA-AM or A-23187 treatment (n = 3 mice). **p** Effects of KB-R7943, BAPTA-AM, and A-23187 treatment on NETs formation in CSE-stimulated neutrophils from human peripheral blood. Scale bar = 2 μm. **q** Pulmonary function parameter $FEV_{20(ms)}$/FVC of Control, KB-R7943, CS, CS+KB-R7943 mice were measured after 12-week CS exposure (n = 6 mice). Each data point represents one biologically independent replicate with 3 technical replicates (**a, c, m, q**). The data shown in the **d-f, j, k, n, o** represent the mean of measurements (3 biological replicates with 3 technical replicates per group). All quantitative data are presented as Mean ± SD. Two-sided *t*-test (**m**) and one-way ANOVA with Tukey's multiple comparison test (**a,**

c, q) were used to calculate the *p* values. At least 3 times each experiment was independently repeated with similar results. Source data are provided as a Source Data file. CSE, cigarette smoke extract; PMA, Phorbol 12-myristate 13-acetate; fMLP, N-Formylmethionyl-leucyl-phenylalanine; $FEV_{20(ms)}$, forced expiratory volume in 20 ms; OE-NCX1, NCX1 overexpression; NC-NCX1, NCX1 negative control; Cit-H3, Citrullinated Histone H3; WT, wild type; min, minutes.

Supplementary Table 1. Clinical characteristics and demographics of clinical specimens.

Variables	Control (n=20)	Emphysema (n=10)	Mixed CBE (n=20)
Age, yrs	70.10±8.84	68.77±9.3	72.70±8.58
Sex,n (%), Male	17 (85.00)	7 (70.00)	18 (90.00)
BMI, kg/m^2	23.64±3.85	20.9±1.84	21.65±2.31
Smokers (%)	12 (60.00)	8 (80.00)	15 (75.00)
Current smokers	6 (30.00)	3 (30.00)	3 (15.00)
Tobacco Exposure (Pack-years)	42.05±22.61	37.9±21.75	49.29±20.65
History of tuberculosis	1 (5.00)	1 (10.00)	1 (5.00)
Diabetes	2 (10.00)	2 (20.00)	1 (5.00)
Systolic blood pressure	130.5±15.17	123.90±16.17	131.7±13.93
Diastolic blood pressure	76.77±7.79	70.9±8.71	75.90±8.60

Supplementary Table 2. Clinical characteristics and demographics of Control and patients with Mixed CBE for BALF and blood collection.

Variables	Control (n=5)	Mixed CBE (n=5)
Age, yrs	58.60±3.05	63±7.78
Sex,n (%), Male	2 (4.00)	4(80.00)
BMI, kg/m^2	23.09±2.55	21.76±2.34
Smokers (%)	2 (40.00)	4 (80.00)
Current smokers	1 (20.00)	2(40.00)
Tobacco exposure (Pack-years)	12.20±17.15	32.8±20.25
History of tuberculosis	0 (0.00)	0 (0.00)
Diabetes	0 (0.00)	1(20.00)
Systolic blood pressure	128.8±9.23	114.8±13.89
Diastolic blood pressure	73.20±4.38	67.2±843

Supplementary Table 3 Primer sequences used in this study.

**Sequences of oligonucleotide primers for PCR**	
Mus-NCX1-F	AGGGACCAAGATGATGAGG
Mus-NCX1-R	GGCTTTTCTGCTGTTGACTT
Mus-MPO-F	ACACCCTCATCCAACCCTT
Mus-MPO-R	ACTTCCAACACGACTCTCC
Mus-NE-F	ATGGCTTGTTTGTGCTGTC
Mus-NE-R	GGCTGATTTAGGTTGGTTC
Human-ncx1-F	TGCTGGTTTGTTCATTTTATTT
Human-ncx1-R	ACCCTGGCTTCTTTCTTACTC
Human-MPO-F	GATGACCCCTGTCTCCTCA
Human-MPO-R	GGTTGTGCTCCCGAAGTAA
Human-NE-F	GCTCTACCCCGATGCCTTT
Human-NE-R	GCCCTTCTCAGTGGGTCCT
Mus-IL-6-F	AGACTTCCATCCAGTTGCCTTC
Mus-IL-6-R	TCTTTTCTCATTTCCACGATTTCCC
Mus-TNF-a-F	ACGCTCTTCTGTCTACTGAACTTC
Mus-TNF-a-R	GTTTGTGAGTGTGAGGGTCTGG
Mus- IL-1β-F	TCTCGCAGCAGCACATCAAC
Mus- IL-1β-R	CCAGCAGGTTATCATCATCATCCC
Mus- CXCL-2-F	CCTCTATTCTGCCAGATGCT
Mus- CXCL-2-R	GCCAGGTTCAGCAGGTAGAC
Mus- IL-17a-F	AGGCCAAGGACTTCCTCCAG
Mus- IL-17a-R	CGGCACTGAGCTTCCCAGAT
Mus-NE-R	
F1: 5’-TGTTAGGAATCATGAGGAGTGCAT-3’	R1: 5’-GCCATAGAAACATTCAGTCAACCC-3’
F2: 5’-AGTCTGAGTTTCAAGATGTGGCTC-3’	R2: 5’-TGACTCCCTGAAAATGAAAAGTCC-3’
**The primer sequences for MRP-Cre:**	
F1: 5’-CATCTGCTGGTTTGGTTATTTGGAG-3’	R1:5'-CTTGCGAACCTCATCACTCGTTG-3’

Supplementary Table 4 Antibody information used in this study.

Application	Antibody target	Supplier name	Catalog No.	Clone name	Dilution
Flow cytometry	PerCP/Cyanine5.5 anti-human CD45	Biolegend	368503	2D1	-
Flow cytometry	PE anti-human CD66b	Biolegend	392903	6/40C	-
Flow cytometry	APC/Fire 750 anti-human CD11b	Biolegend	301351	ICRF44	-
Flow cytometry	Brilliant Violet 605 anti-mouse IgD	Biolegend	405727	11-26c.2a	-
Flow cytometry	CD45-APC-Cy7	BD	557659	30-F11	-
Flow cytometry	CD45-Alexa Fluor 700	Biolegend	147715	I3/2.3	-
Flow cytometry	CD11b-BB515	BD	564454	M1/70	-
Flow cytometry	Ly6G and Ly6C-APC	BD	553129	RB6-8C5	-
Flow cytometry	Ly6G-PE	BD	551461	1A8	-
Flow cytometry	Ly6C-BV605	BD	563011	AL-21	-
Flow cytometry	F4/80-BV421	BD	565411	T45-2342	-
Flow cytometry	Ly6G/Ly6C-FITC	Proteintech	65140	RB6-8C5	-
Flow cytometry	CD11b-APC	Biolegend	101211	M1/70	-
Flow cytometry	CD117(c-kit)-PE	Biolegend	161503	S18020A	-
Western blot	anti–NE	Abclonal	A8953	ARC1364	1:1000
Western blot	anti–MPO	Proteintech	22225-1-AP	polyclonal	1:5000
Western blot	anti–Cit-H3	Abways	CY6587	-	1:5000
Western blot	anti–*Slc8a1*	Abclonal	A5583	-	1:5000
Western blot	anti–β-actin	CST	4967S	-	1:1000
Immunofluorescence	anti-CD11c	Servicebio	GB11059	-	1:200
Immunofluorescence	anti-CD68	Proteintech	25747-1	polyclonal	1:200
Immunofluorescence	anti-CD3	Servicebio	GB11014	-	1:100
Immunofluorescence	anti-Ly-6G	Invitrogen	14-5931-82	RB6-8C5	1:50
Immunofluorescence	anti-αSMA	Invitrogen	50976082	1A4	1:200
Immunofluorescence	anti-CD66b	Novus Biologicals	NB100-77808	G10F5	1:100
Immunofluorescence	anti-SFTPC	Proteintech	10774-1-AP	polyclonal	1:200
Immunofluorescence	anti-MPO	Proteintech	22225-1-AP	polyclonal	1:200

**Application**	**Antibody target**	**Supplier name**	**Catalog No.**	**Clone name**	**Dilution**
Immunofluorescence	anti-NE	Abclonal	A8953	ARC1364	1:200
Immunofluorescence	anti-Cit-H3	Abways	CY6587	-	1:250
Immunofluorescence	anti-NCX1	Abclonal	A5583	-	1:200
