# Supplementary material for: NCX1 reverse mode promotes calcium-dependent Neutrophil Extracellular Trap formation and lung damage in chronic obstructive pulmonary disease
Source: Nat Commun. 2026 Mar 11;17:3801. doi: 10.1038/s41467-026-69636-1 (PMC13111594; doi:10.1038/s41467-026-69636-1)
Supplement: Supplementary file 2 — Reporting Summary [file 41467_2026_69636_MOESM2_ESM.pdf]

nature portfolio

Corresponding author(s): Ting-Hua Wang, Yang Xu, Yao Ouyang

Last updated by author(s): Nov 18, 2025

Reporting Summary

Nature Portfolio wishes to improve the reproducibility of the work that we publish. This form provides structure for consistency and transparency in reporting. For further information on Nature Portfolio policies, see our Editorial Policies and the Editorial Policy Checklist.

Statistics

For all statistical analyses, confirm that the following items are present in the figure legend, table legend, main text, or Methods section.

n/a	Confirmed	
☐	☒	The exact sample size (*n*) for each experimental group/condition, given as a discrete number and unit of measurement
☐	☒	A statement on whether measurements were taken from distinct samples or whether the same sample was measured repeatedly
☐	☒	The statistical test(s) used AND whether they are one- or two-sided<br>*Only common tests should be described solely by name; describe more complex techniques in the Methods section.*
☒	☐	A description of all covariates tested
☐	☒	A description of any assumptions or corrections, such as tests of normality and adjustment for multiple comparisons
☐	☒	A full description of the statistical parameters including central tendency (e.g. means) or other basic estimates (e.g. regression coefficient) AND variation (e.g. standard deviation) or associated estimates of uncertainty (e.g. confidence intervals)
☐	☒	For null hypothesis testing, the test statistic (e.g. *F*, *t*, *r*) with confidence intervals, effect sizes, degrees of freedom and *P* value noted<br>*Give P values as exact values whenever suitable.*
☒	☐	For Bayesian analysis, information on the choice of priors and Markov chain Monte Carlo settings
☒	☐	For hierarchical and complex designs, identification of the appropriate level for tests and full reporting of outcomes
☐	☒	Estimates of effect sizes (e.g. Cohen's *d*, Pearson's *r*), indicating how they were calculated

Our web collection on statistics for biologists contains articles on many of the points above.

Software and code

Policy information about availability of computer code

Data collection	BD FACSDiva (v8.0.l.l) for flow cytometry, DSI BuxcoPFT for pulmonary function test, Zeiss LSM 980 and scanning electron microscopy (ZEISS, EVO10) for image collection, Bio-Rad CFX96 for qPCR, BIO-RAD Gel Doc XR+ for WB.
Data analysis	For flow cytometry: FlowJo (v.10.0).<br>For immuno-staining images quantification: ImageJ_Fiji; Image-Pro Plus.<br>For statistical analysis: SPSS Statistics (v.25.0), Graph Pad PRISM (v.9.0).<br>For graph making: Adobe Illustrator(v.26.2.0).<br>For Calcium flux: imFluxes software (v.3.0), multifunctional microplate detector.

For manuscripts utilizing custom algorithms or software that are central to the research but not yet described in published literature, software must be made available to editors and reviewers. We strongly encourage code deposition in a community repository (e.g. GitHub). See the Nature Portfolio guidelines for submitting code & software for further information.

Data

Policy information about availability of data

All manuscripts must include a data availability statement. This statement should provide the following information, where applicable:

- Accession codes, unique identifiers, or web links for publicly available datasets
- A description of any restrictions on data availability
- For clinical datasets or third party data, please ensure that the statement adheres to our policy

All original data that support the findings of this study are presented as individual values to allow full transparance and are available in Supplementary Information and Source Data file.

Research involving human participants, their data, or biological material

Policy information about studies with human participants or human data. See also policy information about sex, gender (identity/presentation), and sexual orientation and race, ethnicity and racism.

Reporting on sex and gender	Lung specimens in Clinical Biobank of Sichuan University West China Hospital were collected from lung cancer patients (male and female). Bronchoalveolar lavage fluid (BALF) samples were additionally collected from COPD patients and healthy controls (male and female).
Reporting on race, ethnicity, or other socially relevant groupings	This study does not address issues related to race, ethnicity, or other socially relevant groupings.
Population characteristics	Lung specimens were collected from lung cancer patients, and only lung tissues from distal areas far from the cancer site were included in this study. Based on chest computed temography (CT) imaging, samples were stratified into 3 groups: Control group, normal lung tissue from individuals without chronic lung disease; Emphysema group, radiologically confirmed emphysema without chronic bronchitis features; Mixed CBE group, combined features of chronic bronchitis and emphysema. Bronchoalveolar lavage fluid (BALF) samples were additionally collected from COPD patients and healthy controls. COPD patients were diagnosed based on the Global Initiative for Chronic Obstructive Lung Disease (GOLD) guidelines for the diagnosis, management, and prevention of COPD.
Recruitment	Between April and May 2025, 5 healthy individuals (patients with asymptomatic pulmonary nodules) and 5 patients with clinically diagnosed COPD were recruited from the Affiliated Hospital of Zunyi Medical University (Guizhou, China). Among the five healthy controls, four were non-smokers and one was a smoker, while all five COPD patients had a history of smoking.
Ethics oversight	All participants provided written informed consent prior to sample collection. The usage of clinical lung samples from Clinical Biobank of Sichuan University West China Hospital was approved by Science and Technology Review Board and Ethics Committee of Sichuan University (Sichuan, China, Ethics approval: IORG No. 20221582). The BALF sample collection protocol was reviewed and approved by the Ethics Committee of the Affiliated Hospital of Zunyi Medical University (Approval No. KLL-2025-019). This study was conducted in strict accordance with the Declaration of Helsinki Ethical Principles for Medical Research Involving Human Subjects.

Note that full information on the approval of the study protocol must also be provided in the manuscript.

Field-specific reporting

Please select the one below that is the best fit for your research. If you are not sure, read the appropriate sections before making your selection.

☒ Life sciences ☐ Behavioural & social sciences ☐ Ecological, evolutionary & environmental sciences

For a reference copy of the document with all sections, see nature.com/documents/nr-reporting-summary-flat.pdf

Life sciences study design

All studies must disclose on these points even when the disclosure is negative.

Sample size	The clinical sample size was determined by the maximum available clinical at the early time point. Lung tissues from Control patients (n = 20), emphysema patients (n = 10), and Mixed CBE patients (n = 20) were used for immunofluorescence staining, RT-qPCR, WB detection. Additional 5 healthy individuals (patients with asymptomatic pulmonary nodules) and 5 patients with clinically diagnosed COPD were included in this study for bronchoalveolar lavage fluid collection.<br>Animal sample size was determined by existing studies in the field to enable statistical power and reproducibility of results. A minimum of 5–6 biological replicates per experiment were used, with each biological replicate containing 3 technical replicates. The sample sizes were chosen to allow sufficient power for statistical analysis and to avoid underpowered conclusions. No formal statistical method for sample size calculation was applied; however, these numbers are consistent with established standards for animal studies in similar research areas.
Data exclusions	No data was excluded from the relevant analyses.
Replication	Reproducibility was ensured by performing all animal experiments with 5–6 independent biological replicates, each with 3 technical replicates. Key experiments were repeated at least three times independently with similar results, and all attempts at replication were successful. No

	findings reported in this study failed to be replicated.
Randomization	Clinical samples were recruited in strict accordance with the disease criteria, thus the experiments were not randomized. Randomization was not utilized in animal study since Slc8a1 conditional knockout mice were constructed, and the cell products were derived from the same genotype.
Blinding	Experiments were performed by laboratory personnel who were without prior knowledge of the study design.

Reporting for specific materials, systems and methods

We require information from authors about some types of materials, experimental systems and methods used in many studies. Here, indicate whether each material, system or method listed is relevant to your study. If you are not sure if a list item applies to your research, read the appropriate section before selecting a response.

Materials & experimental systems

n/a	Involved in the study	
☐	☒	Antibodies
☒	☐	Eukaryotic cell lines
☒	☐	Palaeontology and archaeology
☐	☒	Animals and other organisms
☒	☐	Clinical data
☒	☐	Dual use research of concern
☒	☐	Plants

Methods

n/a	Involved in the study	
☒	☐	ChIP-seq
☐	☒	Flow cytometry
☒	☐	MRI-based neuroimaging

Antibodies

Antibodies used	Detailed information of primary antibodies was provided in Supplementary Table 3.<br>Flow cytometry antibodies used in this study: PerCP/Cyanine5.5 anti-human CD45 (Biolegend, 368503), PE anti-human CD66b (Biolegend, 392903), APC/Fire 750 anti-human CD11b (Biolegend, 301351), Brilliant Violet 605 anti-mouse IgD (Biolegend, 405727), CD45-APC-Cy7 (BD, 557659), CD45-Alexa Fluor 700 (Biolegend, 147715), CD11b-BB515 (BD, 564454), Ly6G and Ly6C-APC (BD, 553129), Ly6G-PE (BD, 551461), Ly6C-BV605 (BD, 563011), F4/80-BV421 (BD, 565411), Ly6G/Ly6C-FITC (Proteintech, 65140), CD11b-APC (Biolegend, 101211), CD117(c-kit)-PE (Biolegend, 161503).<br>WB antibodies used in this study: anti–NE (Abclonal, A8953), anti–MPO (Proteintech, 22225-1-AP), anti–Cit-H3 (Abways, CY6587), anti–Slc8a1 (Abclonal, A5583), anti–β-actin (CST, 4967S).<br>Immunofluorescence antibodies used in this study: CD11c (Servicebio, GB11059), CD68 (Proteintech, 25747-1), CD3 (Servicebio, GB11014), Ly-6G (Invitrogen, 14-5931-82), αSMA (Invitrogen, 50976082), CD66b (Novus Biologicals, NB100-77808), SFTPC (Proteintech, 10774-1-AP), MPO (Proteintech, 22225-1-AP), NE (Abclonal, A8953), Cit-H3 (Abways, CY6587) and NCX1 (Abclonal, A5583).
Validation	All antibodies used in this study were validated by the manufacturer for antigen specificity and intended use. Validation methods included testing against knockout cell lines for western blotting, immunofluorescence, or FACS, and/or western blotting against recombinant proteins for each specific antigen. Detailed validation statements are available on the manufacturer's websites for each catalog number, which are referenced for the relevant antibodies in the manuscript.

Animals and other research organisms

Policy information about studies involving animals; ARRIVE guidelines recommended for reporting animal research, and Sex and Gender in Research

Laboratory animals	8-12-week-old male Slc8a1flox/flox (Slc8a1f+/f+) and Mrp8 (S100a8) Cre recombinase (Mrp8-Cre) mice, both on a C57BL/6 background, were purchased from Cyagen Biosciences (Beijing, China).
Wild animals	N/A.
Reporting on sex	The study exclusively used male mice due to consistent and reproducible disease progression observed in this group for the COPD model. Male mice are often preferred in this type of research, since androgen levels (such as testosterone) are relatively stable in male mice, whereas estrogen and progesterone in female mice fluctuate cyclically, which may affect the measurement of COPD related indicators such as airway inflammation and mucus secretion. Thus, the variability introduced by sex differences could be minimized. Future studies may investigate potential sex differences in COPD progression using both male and female mice.
Field-collected samples	All mice used in the studies were housed under specific pathogen-free conditions, with controlled temperature, humidity, and a 12-hour light/dark cycle, and were provided with food and water ad libitum.
Ethics oversight	All animal experiments were conducted in strict accordance with the Guidelines for the Care and Use of Laboratory Animals and approved by the Animal Ethics Committee of Zunyi Medical University (Approval No. ZMU20202409).

Note that full information on the approval of the study protocol must also be provided in the manuscript.

Plants

Seed stocks	N/A
Novel plant genotypes	N/A
Authentication	N/A

Flow Cytometry

Plots

Confirm that:

- [x] The axis labels state the marker and fluorochrome used (e.g. CD4-FITC).
- [x] The axis scales are clearly visible. Include numbers along axes only for bottom left plot of group (a 'group' is an analysis of identical markers).
- [x] All plots are contour plots with outliers or pseudocolor plots.
- [x] A numerical value for number of cells or percentage (with statistics) is provided.

Methodology

Sample preparation	Human/mouse BALF: BALF cells were filtered through 100 µm and 70 µm cell strainers to obtain a single-cell suspension. Red blood cell lysis was performed by incubating the suspension with RBC lysis buffer for 5 min at room temperature. The resulting cell pellet was resuspended in 200 µL of FACS buffer.<br>Mouse lung: A digestion cocktail including DNase (50 U/ml, Sigma), Dispase (15 U/ml, Beyotime), and Collagenase Type I (225 U/ml, Servicebio) was tracheally perfused into the mouse lung, followed by perfusion with warm PBS through the right ventricle. The lungs were then subsequently removed from the chest. To prepare single-cell suspensions for FACSanalysis, the lungs were digested using a cocktail consisting of DNase (40 U/ml, Sigma) and Liberase TM (80 mg/mL, Roche), diced with tissue scissors, and further processed using the gentleMACS Octo Dissociator with Heaters (Miltenyibiotec, 130096427). The obtained suspensions were washed with FACS buffer and passed through 70 µm and 40 µm cell filters, respectively. After centrifugation, the precipitation was re-suspended with RBC lysis buffer (Thermo Fisher, NC9067514), incubated for 3 min, centrifuged again, and finally washed twice with FACS buffer to remove residual lysis buffer.
Instrument	The stained cells were analyzed and sorted by BD FACSCanto II (BD, USA).
Software	The data were analyzed using Flow Jo (Version 10.0).
Cell population abundance	The sorted neutrophils was verified with >95-98% purity, which are abundant enough for subsequent analysis.
Gating strategy	Neutrophils were quantified as CD45+CD11b+Gr-1+Ly6g-Ly6g+, and sorted as CD45+CD11b+CD66b+. Non-neutrophils were sorted as as CD45+CD66b-.

- [x] Tick this box to confirm that a figure exemplifying the gating strategy is provided in the Supplementary Information.
